# The relationship between ^18^F-FDG-PETCT-derived tumour metabolic activity, nutritional risk, body composition, systemic inflammation and survival in patients with lung cancer

**DOI:** 10.1038/s41598-020-77269-7

**Published:** 2020-11-30

**Authors:** Ross D. Dolan, John D. Maclay, Tanvir Abbass, David Colville, Fatema Buali, Nicholas MacLeod, Stephen T. McSorley, Paul G. Horgan, Donald C. McMillan

**Affiliations:** 1 Academic Unit of Surgery, School of Medicine, University of Glasgow, Glasgow Royal Infirmary, New Lister Building, Glasgow, G4 0SF UK; 2 Department of Respiratory Medicine, Glasgow Royal Infirmary, Glasgow, G4 0SF UK; 3 West of Scotland PET Centre, Gartnavel Hospital, Tom Wheldon Building, 1053 Great Western Road, Glasgow, G12 0YN UK; 4 Department of Oncology, Beatson West of Scotland Cancer Centre, 1053 Great Western Road, Glasgow, G12 0YN UK

**Keywords:** Cancer imaging, Lung cancer

## Abstract

The aim of this study was to examine the relationship between PET-CT derived tumour glucose uptake as measured by maximum standard glucose uptake (SUVmax) and total lesion glycolysis (TLG), nutritional risk as measured by the malnutrition universal screening tool (MUST), CT derived body composition as measured by skeletal muscle index (SMI) and skeletal muscle radiodensity (SMD), the systemic inflammatory response as measured by the modified Glasgow prognostic score (mGPS) and the neutrophil to lymphocyte ratio (NLR) and survival in patients with lung cancer, treated with radiotherapy. In a retrospective cohort study, 119 patients were included in final analyses. The majority of patients were over 65 (86%), female (52%), had a performance status (ECOG-PS) of 0 or 1 (57%), were at nutritional risk (57%), were overweight (53%), had visceral obesity (62%), had a normal SMI (51%), had a low SMD (62%) and were systemically inflammed (mGPS 1/2, 51%). An elevated TLG was associated with sex (p < 0.05), TNM stage (p < 0.001), MUST (p < 0.01) and mGPS (p < 0.01). An elevated mGPS was associated with age (p < 0.05), NLR (p < 0.01), MUST (p < 0.01), and TLG (p < 0.01). On univariate survival analysis, TNM stage (p < 0.01), mGPS (p < 0.05), NLR (p < 0.01), MUST (p ≤ 0.001), Low SMD (p < 0.05), SUVmax (p ≤ 0.001) and TLG (p < 0.001) were associated with overall survival. On multivariate survival analysis MUST (HR: 1.49 95%CI 1.12–01.98 p < 0.01) and TLG (HR: 2.02 95%CI 1.34–3.04 p = 0.001) remained independently associated with survival. In conclusion, elevated tumour metabolic activity was associated with more advanced stage, greater nutritional risk, the systemic inflammatory response and poorer survival but not body composition analysis in patients with lung cancer. These results suggest that detrimental body composition is not directly determined by tumour metabolic activity but rather an ongoing systemic inflammatory response.

## Introduction

Cancer remains one of the leading causes of mortality worldwide and is responsible for approximately 8.8 million deaths per year^1^. Overall, it has been estimated that one in three people will develop cancer in their lifetime, and one in four will die from it^2,3^. Globally, lung cancer is the most common cancer type and is responsible for 1.69 million deaths per year^1^. In the UK lung cancer is the 3rd most common cancer accounting for 13% of all new cancer cases^4^.

Patients with lung cancer have long been recognized to be at nutritional risk. A widely used method of assessing nutritional risk is the Malnutrition Universal Screening Tool (MUST) which is a five-step screening tool to identify adults who are malnourished and at risk of malnutrition^5^. The relationship between CT defined body composition and outcomes in patients with lung cancer has been widely reported^6^. Loss of skeletal muscle quantity as measured by skeletal muscle index (SMI) and quality as measured by skeletal muscle density (SMD) have both been shown to directly relate to patient morbidity, response to treatment and survival^7–10^. However, the basis of the progressive nutritional and functional decline (often termed cachexia) in these patients is not clear. Historically, this cachexia was considered to be due to the increased metabolic activity of the tumour (including the micro environment). More recently cachexia has been considered as part of disease related malnutrition with inflammation^11^. However, there have been few studies directly comparing tumour and host factors on loss of skeletal muscle in patients with cancer.

It is now possible to examine the metabolic activity of lung tumours with total lesion glycolysis (TLG) using clinically available Positron Emission Tomography (PET) to examine the uptake of glucose using the tracer ^18^F-2-fluoro-2-deoxy-d-glucose (18FDG)^12^. This PET technique, based on the uptake of glucose and combined with CT scanning gives both metabolic and anatomic assessment of the tumour and metastases^13^. PET-CT scanning is used extensively in patients with cancer including lung cancers in clinical decision making as it is widely used to assess disease extent; particularly when other imaging modalities are equivocal. In addition, a recent systematic review by Dolan and co-workers showed that PET-CT tumour metabolic measurements were prognostic in patients with cancer including lung cancer^14^.

In the last decade it has become clear that the systemic inflammatory response (SIR) has prognostic value in both operable and advanced lung cancer^15,16^. In addition, the importance of the systemic inflammatory response as a unifying mechanism for weight loss, loss of lean tissue and poor outcomes in patients with cancer is increasingly recognised^17–19^. Indeed, it has been reported that SMI and SMD are inversely associated with measures of the systemic inflammatory response such as the neutrophil lymphocyte ratio (NLR) and modified Glasgow Prognostic Score (mGPS)^9,20–26^. The NLR is a direct comparative ratio created by dividing the level of neutrophils by lymphocytes from the differential white blood cell count. The mGPS is a cumulative prognostic score constructed from the widely accepted cut of values of > 10 mg/L for CRP and < 35 g/L for albumin. Both the NLR and mGPS have been shown to be prognostic in patients with both operable and inoperable cancers^15,16^.

Since both TLG and mGPS have prognostic value, it is of interest that in a recent systematic review there was a direct relationship between both tumor and bone marrow 18FDG uptake and the systemic inflammatory response^14^. This suggests a potential mechanism of action for the multi-systemic effects of the systemic inflammatory response in patients with cancer^27^. Therefore, it may be hypothesised that high tumour glucose uptake causes both the activation of the systemic inflammatory response and loss of skeletal muscle directly and that this is related to poor patient outcomes. Alternatively, it may also be hypothesised that an elevated tumour glucose uptake is associated with the activation of the systemic inflammatory response and this in turn stimulates the breakdown of skeletal muscle and that this is related to poor clinical outcomes. Therefore, the primary aim of the present study was to examine the relationship between imaging derived tumour glucose uptake, the systemic inflammatory response, nutritional risk and CT-derived body composition in patients with lung cancer. The secondary aim of this study was to assess the impact of these factors on outcomes including survival in patients with lung cancer.

## Material and methods

### Patients

All patients with clinically confirmed non metastatic lung cancer treated with radical radiotherapy in North Glasgow between June 2008 and December 2012, who also underwent staging CT and 18F FDG-PETCT imaging prior to their treatment at the Beatson Oncology Centre, Glasgow were included in the study. All patients were staged using the 7th edition of TNM staging^28^. The dose of radiotherapy varied from 5420 to 6400 cGy with the most common dose being 5500 cGy (62%) and 5400 cGy (35%). Patients had routine blood sampling including a full blood count, serum C-reactive protein (CRP) and albumin concentration at the time of their staging scan. Patients were followed up for 5 years or until death. Ethical approval for this study was granted Greater Manchester East Research Ethics Committee (Rec number: 17/NW/0190). All aspects of this study were performed in accordance with the Declaration of Helsinki.

### Methods

Data were collected prospectively in a database, anonymised and subsequently analyzed including patient demographics, clinicopathological, oncological and radiological data. Body composition CT scan analysis (Dolan RD and Buali F) and 18F FDG-PETCT scan analysis (Colville D) were performed retrospectively by clinicians blinded to clinical outcomes and markers of systemic inflammatory response. Visceral obesity (VO), low SMI (sarcopenia) and low SMD (myosteatosis) were recorded as measures of body composition and measured PET-CT parameters included maximum standardised tumour uptake value (SUVmax), mean standardized tumour uptake (SUVmean) and metabolic tumour volume (MTV)^26,29^. Tumour derived glucose uptake was then calculated as total lesion glycolysis (TLG) using the following formula: TLG = SUVmean × MTV^14,29^.

Serum concentrations of CRP (mg/L) were measured using an autoanalyzer (Architect; Abbot Diagnostics, Maidenhead, UK) with a lower detectable limit of 0.2 mg/L as was serum albumin (normal range 35–50 g/L). Differential blood cell counts were conducted as per local protocols with neutrophil and lymphocyte counts based on those previously reported^30^. The modified Glasgow Prognostic Score (mGPS) was calculated in patients for whom serum CRP and albumin concentrations were available^31^. The neutrophil lymphocyte ratio (NLR) was calculated for each patient for whom neutrophil and lymphocyte counts were available and thresholds were created as previously reported^32^.

### Body composition CT analysis

CT images were obtained at the level of the third lumbar vertebra as previously described^20,33^. Patients who had scans 3 months or more prior to starting radiotherapy were excluded from the study. Scans were excluded if they had significant movement artefacts or were missing region of interest^33^. Each image was analysed using Image J (NIH version 1.47, https://rsbweb.nih.gov/ij/)^20,33^.

Region of interest measurements were made of visceral fat, subcutaneous fat, and skeletal muscle areas (cm^2^) using standard Hounsfield Unit (HU) ranges (adipose tissue − 190 to − 30, and skeletal muscle − 29 to + 150)^20,33^. These were then normalised for height^2^ to create indices; total fat index (TFI, cm^2^/m^2^), subcutaneous fat index (SFI, cm^2^/m^2^), visceral fat index (VFI, cm^2^/m^2^), and skeletal muscle index (SMI, cm^2^/m^2^)^20,33^. Skeletal muscle radiodensity (SMD, HU) was measured from the same region of interest used to calculate SMI, as its mean HU^20,33^. Visceral obesity was defined by Doyle and colleagues as a visceral fat area > 160 cm^2^ for male patients and > 80 cm^2^ for female patients^34^. Sarcopenia was defined by Martin and colleagues as an SMI of < 43 cm^2^/m^2^ if BMI < 25 kg/m^2^ and SMI < 53 cm^2^/m^2^ if BMI ≥ 25 kg/m^2^ in male patients and SMI < 41 cm^2^/m^2^ if BMI < & ≥ 25 kg/m^2^ in female patients^35^. Myosteatosis was defined by Martin and colleagues as an SMD < 41HU in patients with BMI < 25 kg/m^2^ and < 33HU in patients with BMI ≥ 25 kg/m^35^.

Measurements were carried out by two researchers (Dolan RD) and (Buali F). Inter-rater reliability was assessed in 30 test patient images using inter-class correlation coefficients (Total fat area = 1.000, Subcutaneous fat area = 1.000, Visceral fat area = 1.000, Skeletal muscle area = 0.986, Skeletal muscle density = 0.974). Investigators were blind to patient’s demographic and clinico-pathological status.

### 18F FDG-PETCT

18F FDG-PETCT scanning was performed in accordance with departmental standard procedures based on the EANM guidelines^36^ on one of the two multimodality PETCT scanners (Discovery-690 or 710, General Electric System, Milwaukee, WI, USA). Patients were fasted for 6 h prior to and 1 h after the IV injection of 400 MBq 18F-FDG^29^. The level of blood glucose was measured before 18F-FDG injection to guarantee concentrations < 11 mmol/l. Initial unenhanced CT images were acquired using a 120 kV automatic mA modulation range of 15–240 mAs^29^. The torso CT covered from the skull base to the mid-thigh and was reconstructed at 2.5 mm increments^29^. This was followed by PET images, encompassing the same transverse field of view as the CT. PET acquisition time was 3–4 min per bed position^29^. PET attenuation correction was based on the CT data and images were corrected for scatter and iteratively reconstructed using Time of Flight and SharpIR on a 192 × 192 matrix^29^.

PETCT images were analysed on GE Advantage Workstation using a SUVmax of 7 g/ml threshold level to view the PET images^29^. SUVmean and MTV were obtained from 3D isocontour at 42% of the maximal pixel value (VOL42)^29^. TLG was calculated according to the following formula: TLG = SUVmean × MTV. PETCT data were measured from the region of interest (ROI) placed over the dominant sites^14,29^.

### Statistical analysis

ROC curve analysis determined the optimum thresholds for SUVmax and TLG. Body composition and PET-CT measurements were presented as median and range and compared using Mann–Whitney or Kruskal–Wallis tests^26,33,37^. Categorical variables were analysed using χ2 test for linear-by-linear association, or χ^2^ test for 2 by 2 tables^26,33,37^.

Univariate and multivariate survival data were analysed using Cox’s proportional hazards model^26,33^. Variables associated with overall survival at a significance level of p < 0.1 on univariate analysis were included in multivariate modeling using backward conditional regression where a two-sided p value < 0.05 was considered statistically significant^26,33^. Overall survival was defined as time from date of 18F FDG-PETCT to date of death due to any cause^26,33^. P values < 0.05 were considered statistically significant^26,33^. Statistical analysis was performed using SPSS software (Version 21.0. SPSS Inc., Chicago, IL, USA)^26,33^.

### Ethical approval

Ethical approval for this study was granted Greater Manchester East Research Ethics Committee (Rec number: 17/NW/0190). These was a retrospective observational cohort study with no change in patient management. As a result, informed consent was not required in accordance with ethical approval. However, as per standard clinical practice in Greater Glasgow and Clyde informed consent to take part in clinical research was taken prior to commencing radiotherapy. All aspects of this study were performed in accordance with the Declaration of Helsinki.

## Results

In total, 251 patients were identified as having undergone potentially curative radiotherapy for lung cancer. Of these, 61 were excluded due to scanning taking place more than 3 months before commencing radiotherapy. A further 71 patients were excluded due to the absence of markers of the systemic inflammatory response, CT derived body composition measurements or a histological diagnosis of small cell lung cancer (SCLC). A total of 119 patients (57 males, 62 females) were included in final analyses. The relationship between clinicopathological characteristics, tumour activity, body composition, markers of the systemic inflammatory response and overall survival are shown in Table 1. The majority of patients were over 65 (86%), female (52%), had a performance status (ECOG-PS) of 0 or 1 (57%), were at nutritional risk (57%), were overweight (53%), had visceral obesity (62%), had a normal SMI (51%), had a low SMD (62%) and were systemically inflammed (mGPS 1/2, 51%). All patients were treated with radiotherapy, six patients received additional chemotherapy with two received concurrent chemoradiotherapy. The majority of patients had an elevated TLG (61%) and on follow-up, 107 (90%) patients died and the median survival was 22 months (range 3–91 months). On univariate survival analysis, TNM stage (p < 0.01), mGPS (p < 0.05), NLR (p < 0.01), Low SMD (p < 0.05), SUVmax (p < 0.01) and TLG (p < 0.001) were associated with overall survival (Table 1).

**Table 1:** The relationship between clinicopathological characteristics, tumour activity, body composition, markers of the systemic inflammatory response and overall survival in patients with lung cancer.

Characteristics	n = 119 (%)	Univariate Cox regression analysis OS	*p*-value
**Sex**			
Male	57 (47.9)	1.34 (0.91–1.97)	0.141
Female	62 (52.1)		
**Age**			
< 65	17 (14.3)	1.04 (0.79–1.37)	0.768
65–74	54 (45.4)		
> 75	48 (40.3)		
**TNM**			
I	42 (35.3)	1.40 (1.12–1.74)	0.003
II	22 (18.5)		
III	55 (46.2)		
**ECOG-PS**			
0/1	68 (57.1)	0.74 (0.50–1.09)	0.126
≥ 2	51 (42.9)		
**Inflammatory response**			
mGPS			
0	58 (48.7)	1.30 (1.06–1.61)	0.014
1	20 (16.8)		
2	41 (34.5)		
**NLR**			
< 3	53 (44.5)	1.38 (1.09–1.76)	0.009
3–5	35 (29.4)		
> 5	31 (26.1)		
**MUST**			
Low risk	51 (42.9)	1.60 (1.21–2.11)	0.001
Intermediate risk	52 (43.7)		
High risk	16 (13.4)		
**Body composition**			
BMI kg/m^2^			
<25	56 (47.1)	0.77 (0.54–1.13)	0.182
≥ 25	63 (52.9)		
**Visceral obesity**			
VFA	134.23 (14.35–577.08)	1.00 (0.99–1.01)	0.780
**Visceral obesity**			
No	45 (37.8)	0.81 (0.55–1.20)	0.292
Yes	74 (62.2)		
**Sarcopenia**			
SMI	44.23 (29.40–74.36)	1.00 (0.98–1.02)	0.899
**Low SMI**			
No	61 (51.3)	0.98 (0.67–1.44)	0.930
Yes	58 (48.7)		
**Myosteatosis**			
SMD	34.53 (9.58–51.24)	1.03 (1.00–1.05)	0.043
**Low SMD**			
No	45 (37.8)	0.66 (0.44–0.97)	0.035
Yes	74 (62.2)		
**PET-CT analysis**			
SUVmax	14.60 (3.10–36.9.)	1.03 (1.01–1.06)	0.010
**SUVmax > 11.40**			
No	44 (37.0)	2.05 (1.34–3.14)	0.001
Yes	75 (63.0)		
TLG	102.66 (3.47–2070.90)	1.01 (1.00–1.02)	< 0.001
**TLG > 68.89**			
No	47 (29.5)	2.18 (1.46–3.26)	< 0.001
Yes	72 (60.5)		

*TNM* Tumour, Node, Metastasis, *ECOG-PS* Eastern Cooperative Oncology Group Performance Status, *mGPS* modified Glasgow Prognostic Score, *NLR* Neutrophil Lymphocyte Ratio, *MUST* Malnutrition Universal Screening Tool, *BMI* Body Mass Index, *VFA* Visceral Fat Area, *SMI* Skeletal Muscle Index, *SMD* Skeletal Muscle Density, *TLG* Total Lesion Glycolysis, *SUVmax* Standardised Tumour Uptake Value, *TLG* Total Lesion Glycolysis.

The relationship between the tumour metabolic activity as measured by TLG (≤ 68.89/ > 68.89) and clinicopathological characteristics in patients lung cancer are shown in Table 2. TLG was significantly associated with sex (p < 0.05), TNM stage (p < 0.001), mGPS (p < 0.01), and survival (p < 0.01).

**Table 2:** The relationship between TLG and clinicopathological characteristics in patients with lung cancer.

Characteristics	Low TLG (n = 47)	High TLG (n = 72)	*p*-value
**Sex**			
Male	29 (61.7)	28 (38.9)	0.015
Female	18 (38.3)	44 (61.1)	
**Age**			
< 65	5 (10.60	12 (16.7)	0.578
65–74	21 (44.7)	33 (45.8)	
> 75	21 (44.7)	27 (37.5)	
**TNM**			
I	27 (57.4)	15 (20.8)	< 0.001
II	8 (17.0)	14 (19.4)	
III	12 (25.5)	43 (59.7)	
**ECOG-PS**			
0/1	28 (59.6)	40 (55.6)	0.665
≥ 2	19 (40.4)	32 (44.4)	
**Inflammatory response**			
mGPS			
0	31 (66.0)	27 (37.5)	0.006
1	7 (14.9)	13 (18.1)	
2	9 (19.1)	32 (44.4)	
**NLR**			
< 3	26 (55.3)	27 (37.5)	0.146
3–5	12 (25.5)	23 (31.9)	
> 5	9 (19.1)	22 (30.6)	
**MUST**			
Low risk	29 (61.7)	22 (30.6)	0.003
Intermediate risk	15 (31.9)	37 (51.4)	
High risk	3 (6.4)	13 (18.1)	
**Body composition**			
BMI kg/m^2^			
< 25	19 (40.4)	37 (51.4)	0.241
≥ 25	28 (59.6)	35 (48.6)	
**Visceral obesity**			
VFA	128.94 (15.33–577.08)	140.19 (14.35–549.90)	0.683
**Visceral obesity**			
No	17 (36.2)	28 (38.9)	0.765
Yes	30 (63.8)	44 (61.1)	
**SMI**	43.34 (29.43–66.36)	45.35 (29.40–74.36)	0.350
**Low SMI**			
No	24 (51.1)	37 (51.4)	0.972
Yes	23 (48.9)	35 (48.6)	
**SMD**	31.80 (9.58–48.04)	35.31 (13.98–51.24)	0.098
**Low SMD**			
No	15 (31.9)	30 (41.7)	0.284
Yes	32 (68.1)	42 (58.3)	
**Survival**			
Survival rate (3 year)			
No	26 (55.3)	58 (80.6)	0.003
Yes	21 (44.7)	14 (19.4)	

*TNM* Tumour, Node, Metastasis, *ECOG-PS* Eastern Cooperative Oncology Group Performance Status, *mGPS* modified Glasgow Prognostic Score, *NLR* Neutrophil Lymphocyte Ratio, *MUST* Malnutrition Universal Screening Tool, *BMI* Body Mass Index, *VFA* Visceral Fat Area, *SMI* Skeletal Muscle Index, *SMD* Skeletal Muscle Density, *TLG* Total Lesion Glycolysis.

The relationship between the systemic inflammatory response as measured by mGPS and clinicopathological characteristics in patients lung cancer are shown in Table 3. An elevated mGPS was associated with age (p < 0.05), NLR (p < 0.01), MUST (p < 0.01) and TLG (p < 0.01).

**Table 3:** The relationship between mGPS and clinicopathological/PET-CT characteristics in patients with lung cancer.

Characteristics	mGPS 0 (n = 58)	mGPS 1 (n = 20)	mGPS 2 (n = 41)	*p*-value
**Sex**				
Male	36 (62.1)	7 (35.0)	14 (34.1)	0.617
Female	22 (37.9)	13 (65.0)	27 (65.9)	
**Age**				
< 65	10 (17.2)	4 (20.0)	3 (7.3)	0.011
65–74	25 (43.1)	9 (45.0)	20 (48.8)	
> 75	23 (39.7)	7 (35.0)	18 (43.9)	
**TNM**				
I	23 (39.7)	8 (40.0)	11 (26.8)	0.695
II	11 (19.0)	3 (15.0)	8 (19.5)	
III	24 (41.4)	9 (45.0)	22 (53.7)	
**ECOG-PS**				
0/1	32 (55.2)	13 (65.0)	23 (56.1)	0.735
≥ 2	26 (44.8)	7 (35.0)	18 (43.9)	
**Inflammatory response**				
NLR				
< 3	35 (60.3)	6 (30.0)	12 (29.3)	0.002
3–5	17 (29.3)	6 (30.0)	12 (29.3)	
> 5	6 (10.3)	8 (40.0)	17 (41.5)	
**MUST**				
Low risk	30 (51.7)	11 (55.0)	10 (24.4)	0.007
Intermediate risk	25 (43.1)	7 (35.0)	20 (48.8)	
High risk	3 (5.2)	2 (10.0)	11 (26.8)	
**Body composition**				
BMI kg/m^2^				
< 25	25 (43.1)	8 (40.0)	23 (56.1)	0.348
≥ 25	33 (56.9)	12 (60.0)	18 (43.9)	
**Visceral obesity**				
VFA	122.56 (21.73–549.90)	140.19 (46.71–577.08)	148.45 (14.35–433.30)	0.770
**Visceral obesity**				
No	19 (32.8)	7 (35.0)	19 (46.3)	0.374
Yes	39 (67.2)	13 (65.0)	22 (53.7)	
**SMI**	43.87 (29.40–72.04)	43.80 (29.43–74.36)	44.66 (34.70–73.49)	0.675
**Low SMI**				
No	29 (50.0)	11 (55.0)	21 (51.2)	0.928
Yes	29 (50.0)	9 (45.0)	20 (48.8)	
**SMD**	33.23 (13.98–51.24)	36.65 (9.58–49.96)	35.18 (16.81–49.25)	0.312
**Low SMD**				
No	18 (31.0)	9 (45.0)	18 (43.9)	0.330
Yes	40 (69.0)	11 (55.0)	23 (56.1)	
**PET-CT analysis**				
TLG	64.01 (3.47–912.18)	115.77 (5.15–968.76)	227.40 (10.82–2070.90)	< 0.001
**TLG > 68.89**				
No	31 (53.4)	7 (35.0)	9 (22.0)	0.006
Yes	27 (46.6)	13 (65.0)	32 (78.0)	
**Survival**				
Survival rate (3 year)				
No	37 (63.8)	16 (80.0)	31 (75.6)	0.267
Yes	21 (36.2)	4 (20.0)	10 (24.4)	

*TNM* Tumour, Node, Metastasis, *ECOG-PS* Eastern Cooperative Oncology Group Performance Status, *mGPS* modified Glasgow Prognostic Score, *NLR* Neutrophil Lymphocyte Ratio, *MUST* Malnutrition Universal Screening Tool, *BMI* Body Mass Index, *VFA* Visceral Fat Area, *SMI* Skeletal Muscle Index, *SMD* Skeletal Muscle Density, *TLG* Total Lesion Glycolysis.

The relationship between clinicopathological characteristics, tumour activity, body composition, markers of the systemic inflammatory response and overall survival in patients with lung cancer is shown in Table 4. On univariate survival analysis, TNM stage (p < 0.01), mGPS (p < 0.05), NLR (p < 0.01), MUST (p ≤ 0.001), Low SMD (p < 0.05), SUVmax (p ≤ 0.001) and TLG (p < 0.001) were associated with overall survival. On multivariate survival analysis MUST (HR:1.49, 95%CI 1.12–1.98, p < 0.01), and TLG > 68.89 (HR:2.02, 95%CI 1.34–3.04, p < 0.001) remained independently associated with overall survival.

**Table 4:** The relationship between clinicopathological characteristics, tumour activity, body composition, markers of the systemic inflammatory response and overall survival in patients with lung cancer: Univariate and multivariate analysis.

Characteristics	n = 119 (%)	Univariate Cox regression analysis OS	*p*-value	Multivariate Cox regression analysis OS	*p*-value
**Sex**					
Male	57 (47.9)	1.34 (0.91–1.97)	0.141	**–**	**–**
Female	62 (52.1)				
Age					
< 65	17 (14.3)	1.04 (0.79–1.37)	0.768	**–**	**–**
65–74	54 (45.4)				
> 75	48 (40.3)				
**TNM**					
I	42 (35.3)	1.40 (1.12–1.74)	0.003	**–**	0.329
II	22 (18.5)				
III	55 (46.2)				
**ECOG-PS**					
0/1	68 (57.1)	0.74 (0.50–1.09)	0.126	**–**	**–**
≥ 2	51 (42.9)				
**Inflammatory response**					
mGPS					
0	58 (48.7)	1.30 (1.06–1.61)	0.014	**–**	0.326
1	20 (16.8)				
2	41 (34.5)				
**MUST**					
Low risk	51 (42.9)	1.60 (1.21–2.11)	0.001	1.49 (1.12–1.98)	0.006
Intermediate risk	52 (43.7)				
High risk	16 (13.4)				
**Body composition**					
BMI kg/m^2^					
< 25	56 (47.1)	0.77 (0.54–1.13)	0.182	**–**	**–**
≥ 25	63 (52.9)				
**Visceral obesity**					
No	45 (37.8)	0.81 (0.55–1.20)	0.292	**–**	**–**
Yes	74 (62.2)				
**Sarcopenia**					
Low SMI					
No	61 (51.3)	0.98 (0.67–1.44)	0.930	**–**	**–**
Yes	58 (48.7)				
**Myosteatosis**					
Low SMD					
No	45 (37.8)	0.66 (0.44–0.97)	0.035	**–**	0.192
Y es	74 (62.2)				
**PET-CT analysis**					
TLG > 68.89					
No	47 (29.5)	2.18 (1.46–3.26)	< 0.001	2.02 (1.34–3.04)	0.001
Yes	72 (60.5)				

*TNM* Tumour, Node, Metastasis, *ECOG-PS* Eastern Cooperative Oncology Group Performance Status, *mGPS* modified Glasgow Prognostic Score, *MUST* Malnutrition Universal Screening Tool, *BMI* Body Mass Index, *SMI* Skeletal Muscle Index, *SMD* Skeletal Muscle Density, *TLG* Total Lesion Glycolysis.

## Discussion

The results of the present study show that, in a cohort of patients with lung cancer undergoing radical radiotherapy, there was a significant association between TLG (metabolic activity) and the mGPS (systemic inflammatory response) and both were significantly associated with nutritional risk and survival. However, neither TLG nor mGPS was significantly associated with CT derived body composition. Therefore, in this cross sectional study it is clear that elevated tumour metabolic activity and the systemic inflammatory responses are directly linked but their comparative link to body composition (in particular skeletal muscle mass) requires further investigation.

The results of the present study are consistent with a recent systematic review which reported a relationship between markers of the systemic inflammatory response and PET-CT parameters^14^. However, there was not a significant association between either TLG or mGPS and SMI (skeletal muscle mass). The former relationship has not, to our knowledge, been previously examined in cancer patients. However, the latter relationship between an elevated systemic inflammatory response and low SMI has been reported repeatedly^38^. Therefore, it may be that, given that approximately 50% of patients had a low SMI, the present study was too small to detect a differential effect of TLG and mGPS on low SMI. Further work on larger cancer datasets will be required to determine whether there is indeed a differential effect.

Although the results of the present study do confirm the relationship between TLG and measures of the systemic inflammatory response. The mechanism by which a metabolically active tumour evokes a systemic inflammatory response is not clear. However, in the absence of data on the relationship between an elevated TLG and detailed tumour phenotypying, there are a number of plausible mechanisms. Tumour hypoxia and necrosis and the subsequent production of lactate result in the local activation of innate immune cells and production of pro-inflammatory cytokines, including interleukin-6 (IL-6), stimulating production of CRP^39,40^. Circulating IL-6 levels are linked to tumour necrosis and both local and systemic inflammatory responses in patients undergoing resection for colorectal cancer^39^. An alternative hypothesis is that circulating tumour cells activate myeloid cells in the bone marrow to produce such pro-inflammatory cytokines, in particular IL-6^40^. Indeed, there is some evidence from PET-CT studies that there is increased uptake of glucose from the bone marrow and that the SUVmax from the bone marrow is also associated with markers of the systemic inflammatory response^14^. In the present study, glucose uptake was only examined in the bulk tumour. Irrespective, both of these mechanisms would, in turn, result in a progressive catabolic state with subsequent breakdown of skeletal muscle resulting in cancer related cachexia. 

The results of the present study are also consistent with the proposal of McAllister and Weinberg that the systemic inflammatory response is the tip of the cancer iceberg reflecting tumour/host immune cytokine activity, disordered metabolism and the development of cancer associated symptoms such as loss of appetite, fatigue and poor physical function^41–43^. Given the present results and the increasing importance of the inflammatory responses in the assessment and treatment of lung cancer, it will also be of considerable interest to better define the relationship between tumour metabolic activity and the components of the tumour microenvironment including tumour inflammatory cell infiltrate^44,45^, the tumour stroma^46,47^ and tumour mutational burden measured with circulating tumour DNA .

The present study had a number of limitations including that the data was retrospectively analysed from a prospective audit of clinical practice, the majority of patients were treated with radiotherapy in isolation (97%). This study predates the SOCCAR study of 2014, therefore standard practice was for patients to receive radiotherapy initially followed by chemotherapy depending on their physiological reserve^48^. As a result, just six patients received combined chemo and radiotherapy. Also, histological tumour type was not determined in 21% of cases due to concurrent comorbidities and therefore the present cohort may be a relatively heterogeneous group. However, the present study also has a number of strengths. To our knowledge, this is the first study to comprehensively examine the nature of the relationship between tumour metabolic activity, body composition, the systemic inflammatory response and survival in patients with cancer. The measurements were carried out within one month of each other and the sample size compares favourably to previous studies in the field^14^. Indeed, given the routine clinical measurements used in the present study these results could be readily validated to give a new insight into these relationships in patients with cancer.

The results showing a relationship between the systemic inflammatory response and tumour metabolic activity but not between tumour metabolic activity and CT-derived body composition have some important implications for ongoing patient care. These results suggest that targeting the systemic inflammatory response with the use of anti-inflammatory agents could potentiate the effectiveness of treatments directly targeting the tumour such as surgery or curative radiotherapy^40^. In this way the incorporation of modulation of the systemic inflammatory response may have a profound impact on patient care moving forward.

In conclusion, in patients with lung cancer treated with radical radiotherapy, tumour glucose uptake was associated with activation of systemic inflammatory response and mortality but not lower skeletal muscle mass.

## Conclusion

In patients with lung cancer treated with radical radiotherapy, tumour glucose uptake was associated with activation of systemic inflammatory response and mortality but not lower skeletal muscle mass.
